# Tyrosine kinase inhibitor induced growth factor receptor upregulation enhances the efficacy of near-infrared targeted photodynamic therapy in esophageal adenocarcinoma cell lines

**DOI:** 10.18632/oncotarget.16165

**Published:** 2017-03-13

**Authors:** Elmire Hartmans, Matthijs D. Linssen, Claire Sikkens, Afra Levens, Max J.H. Witjes, Gooitzen M. van Dam, Wouter B. Nagengast

**Affiliations:** ^1^ Department of Gastroenterology and Hepatology, University of Groningen, University Medical Centre, Groningen, The Netherlands; ^2^ Department of Clinical Pharmacy and Pharmacology, University of Groningen, University Medical Centre, Groningen, The Netherlands; ^3^ Department of Oral and Maxillofacial Surgery, University of Groningen, University Medical Centre, Groningen, The Netherlands; ^4^ Department of Surgery, Nuclear Medicine and Molecular imaging and Intensive Care, University of Groningen, University Medical Centre, Groningen, The Netherlands

**Keywords:** targeted photodynamic therapy, esophageal cancer, epidermal growth factor receptor family (HER-family), targeted treatment

## Abstract

Esophageal carcinoma (EC) is a global health problem, with disappointing 5-year survival rates of only 15–25%. Near-infrared targeted photodynamic therapy (NIR-tPDT) is a novel strategy in which cancer-targeted phototoxicity is able to selectively treat malignant cells. In this *in vitro* report we demonstrate the applicability of antibody-based NIR-tPDT in esophageal adenocarcinoma (EAC), using the phototoxic compounds cetuximab-IRDye700DX and trastuzumab-IRDye700DX, targeting respectively epidermal growth factor receptor 1 (EGFR) and 2 (HER2). Furthermore, we demonstrate that NIR-tPDT can be made more effective by tyrosine kinase inhibitor (TKI) induced growth receptor upregulation. Together, these results unveil a novel strategy for non-invasive EAC treatment, and by pretreatment-induced receptor upregulation its future clinical application may be optimized.

## INTRODUCTION

Esophageal cancer (EC) is a global health problem. EC is the sixth leading cause of cancer related mortality and the eighth most commonly diagnosed cancer in the world [1]. EC can be roughly divided into two cancer types: squamous cell carcinoma (SCC), of which the incidence rates have been decreasing, and esophageal adenocarcinoma (EAC). Over the past few decades risk factors such as obesity and gastroesophageal reflux disease (GERD), which are both progressive problems of the Western countries, have caused the incidence rates of EAC to increase [2]. In addition, the current prognosis of EC remains poor, resulting in 5-year survival rates of only 15–25% [3].

Detection and treatment of EAC in an early stage of the disease is essential to improve its outcome [4]. Surgical resection is an invasive treatment option and carries a high risk for morbidity and mortality [5,6]. When EAC is in its limited disease-stage, superficial and still restricted to the mucosa, non-invasive endoscopic resection is the treatment of choice [7]. Though, to optimally treat early stage EAC with positive margins after endoscopic resection, additive treatment approaches are needed. A possible ablative approach is photodynamic therapy (PDT). PDT is able to generate cell death via non-ionizing light energy that interacts with photosensitizers, leading to chemical destruction of cells. Previous research has focused on implementing PDT as an alternative treatment option for early stage EAC and dysplastic Barrett Esophagus (BE) [8]. Although PDT ablation has shown to decrease the development of EAC, considerable side effects such as photo cutaneous reactions and esophageal strictures have also been described [9–11]. Therefore, to date, PDT is considered an effective salvage treatment option for patients who are not fit for surgery, rather than as a first choice of treatment in early EAC or dysplastic BE in general [12].

**Figure 1:**
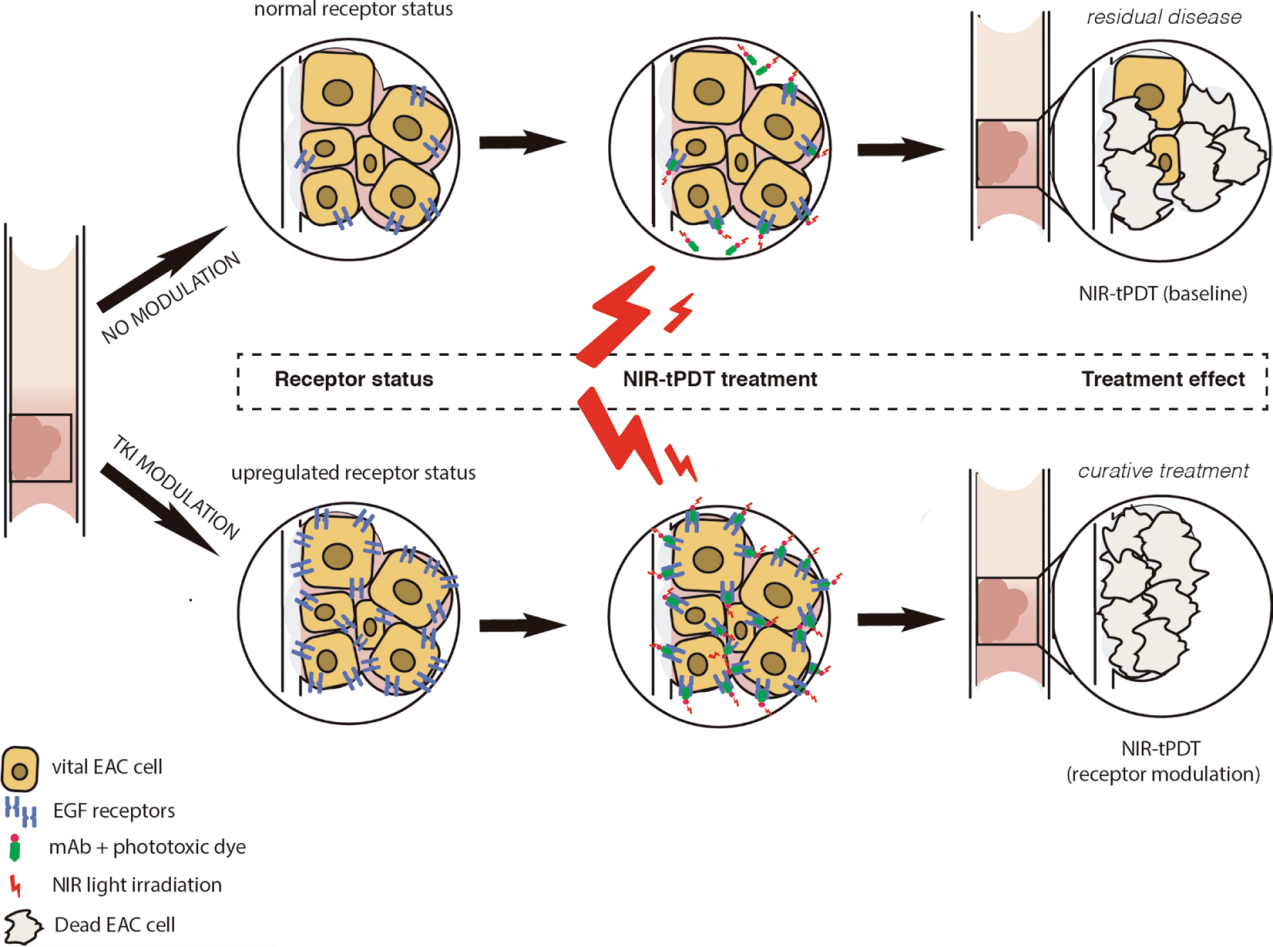
Schematic overview of NIR-tPDT treatment Following TKI pretreatment the receptor status of the EAC cells is modulated. Hypothetically, more receptors will be expressed per cell and more cells will express the receptor at their cell surface as a result of the TKI-induced positive feedback loop. Since more phototoxic conjugate is able to bind to the target cells after modulation, the therapeutic effect of NIR-tPDT will be enhanced. EAC, esophageal adenocarcinoma; NIR-tPDT, near-infrared targeted photodynamic therapy; TKI, tyrosine kinase inhibitor; EGF, epidermal growth factor.

To create a more cancer-selective PDT approach, a new modality known as *targeted* photodynamic therapy (tPDT) has recently been developed [13]. In tPDT cancer-targeting agents, such as monoclonal antibodies (mAbs), are functionalized with a photosensitizer group to form a phototoxic compound. The targeted photosensitizer accumulates in tissue where the marker is expressed, thereby reducing the PDT exposure of the surrounding healthy tissue to a minimum. In this report we are the first to evaluate the application of mAb-based near-infrared (NIR) tPDT in EAC. We make use of mAbs directed against two receptors of the epidermal growth factor (EGF/ErbB) family, EGFR (ErbB1) and HER2 (ErbB2), which have been described to be overexpressed in various solid cancer types, including EC [14–21]. Since the receptor density varies between tumor types as well as between different areas in the same tumor, effectiveness of NIR-tPDT throughout the tumor could be impaired. We hypothesize that when an increased amount of receptors is available at the cell surface, more cancer-targeted phototoxic compound can bind the cancer cells, subsequently improving the therapeutic effect of tPDT (Figure 1). Since tyrosine kinase inhibitors (TKIs) can activate a positive feedback-loop by blocking downstream signaling, they can generate overexpression of the EGF-receptors on the surface of the cell [16, 22–24]. This report evaluates the therapeutic effect of cetuximab (anti-EGFR) and trastuzumab (anti-HER2) targeted NIR-tPDT in Esophageal adenocarcinoma *in vitro* and, as a first, describes modulation of the EGFR and HER2 receptors with use of TKIs, as a tool to enhance the therapeutic effect and applicability of NIR-tPDT.

## RESULTS

### EGFR and HER2 receptor modulation

### Characterization of FLO-1 / OE33: baseline expression of EGFR and HER2 receptors

**Figure 2:**
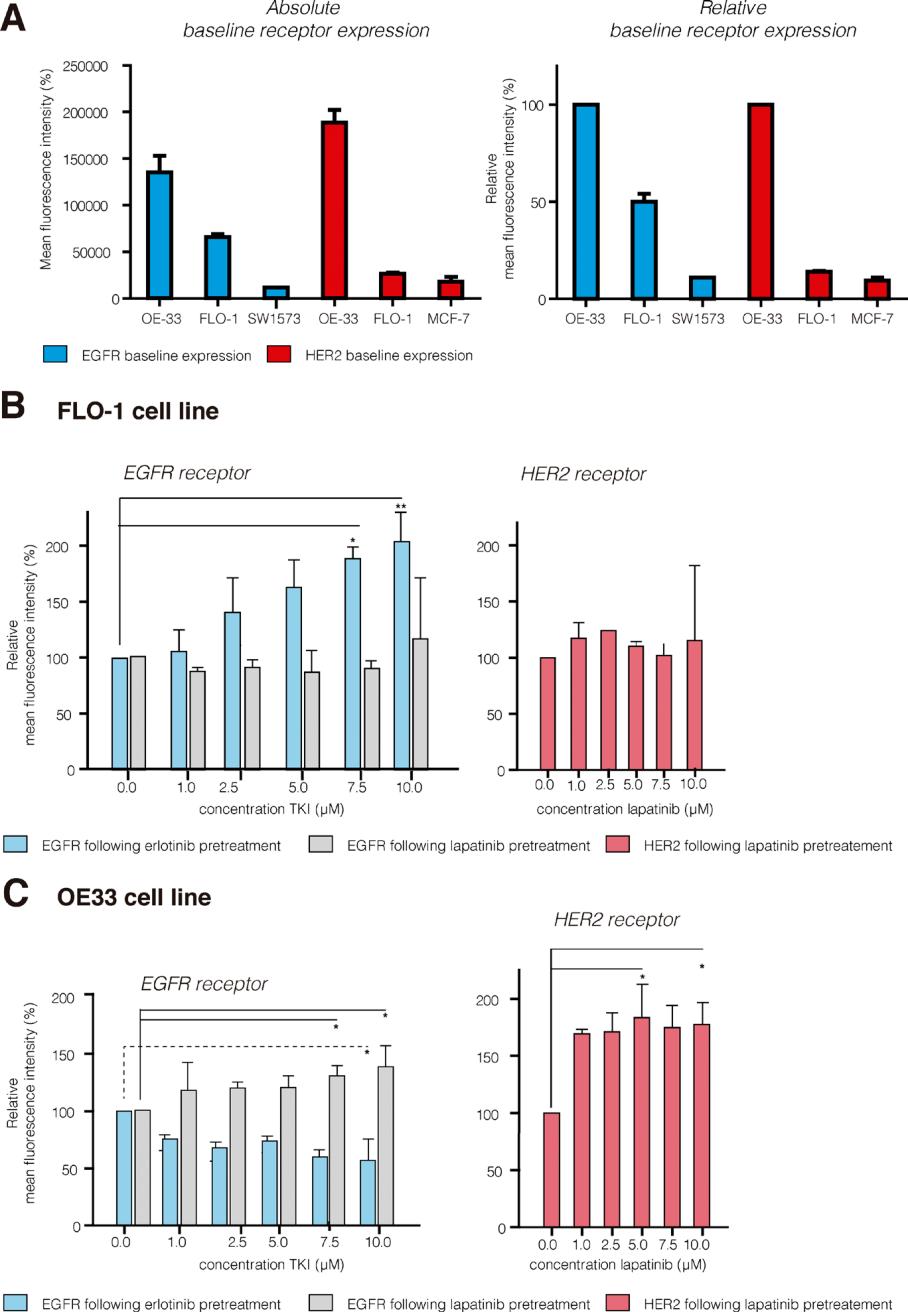
EGFR and HER2 receptor expression results (**A**) Absolute and relative baseline receptor results for the EAC (FLO-1/OE33) and control cell lines (sw1573 and MCF-7), illustrating that the OE33 are high EGFR (blue) and high HER2 (red), and FLO-1 intermediate EGFR and low to negative HER2 expressing cells. (**B**) For FLO-1 pretreatment with erlotinib resulted in significant upregulation of EGFR expression (+104%, 10 μM); EGFR expression significantly decreased in the OE33 cells (−43%, dashed line). (**C**) For OE33 lapatinib pretreatment resulted in significant upregulation of both EGFR (+33%) and HER2 (+77%; 10 μM). For FLO-1 no evident upregulation of HER2 was observed. TKI, tyrosine kinase inhibitor. Results are presented using the mean (SD); *P < 0.05; **P < 0.001.

Figure 2A depicts the baseline EGFR and HER2 receptor baseline expression results for the two EAC cell lines and the growth receptor control cell lines (SW1573 and MCF-7). Both EAC cell lines express EGFR and HER2 (Absolute mean MFI values: OE33 EGFR = 135,814 / HER2 = 188,608; FL0–1 EGFR = 65,854 / HER2 = 26,528). The relative MFI values show that the OE33 cells have high expression of both the EGFR and HER2 receptor. In contrast, the FLO-1 cells show a moderate EGFR and low to negative HER2 expression; when set against the OE33 cells (100%), the FLO-1 cells show 50% EGFR and only 14% HER2 expression.

### TKI treatment for 72 hours induces significant up-regulation of receptor expression

From the different time points tested, 72 hours of lapatinib or erlotinib pretreatment was found to be the most effective for receptor upregulation (Supplementary Figure 1). In the FLO-1 cells, 72 hours of erlotinib pretreatment induced a significant dose-dependent upregulation of EGFR (max. +104%, *P* < 0.001; Figure 2B). The effect of 72 hours of lapatinib pretreatment on the EGFR and HER2 receptors varied greatly between the experiments; overall, a slight increase in receptor expression was seen, but due to the wide variation in results this difference was not significant (EGFR +15%, *P* < 0.5830 and HER2 +15%, *P* < 0.0616). In the OE33 cells 72 hours of erlotinib induced a significant down-regulation of EGFR expression (−43%, *P* < 0.0024; Figure 2C). In contrast, 72 hours of lapatinib induced a significant upregulation of both EGFR and HER2 receptor expression (EGFR +31%, *P* < 0.004 and HER2 +77%, *P* < 0.0046).

**Figure 3:**
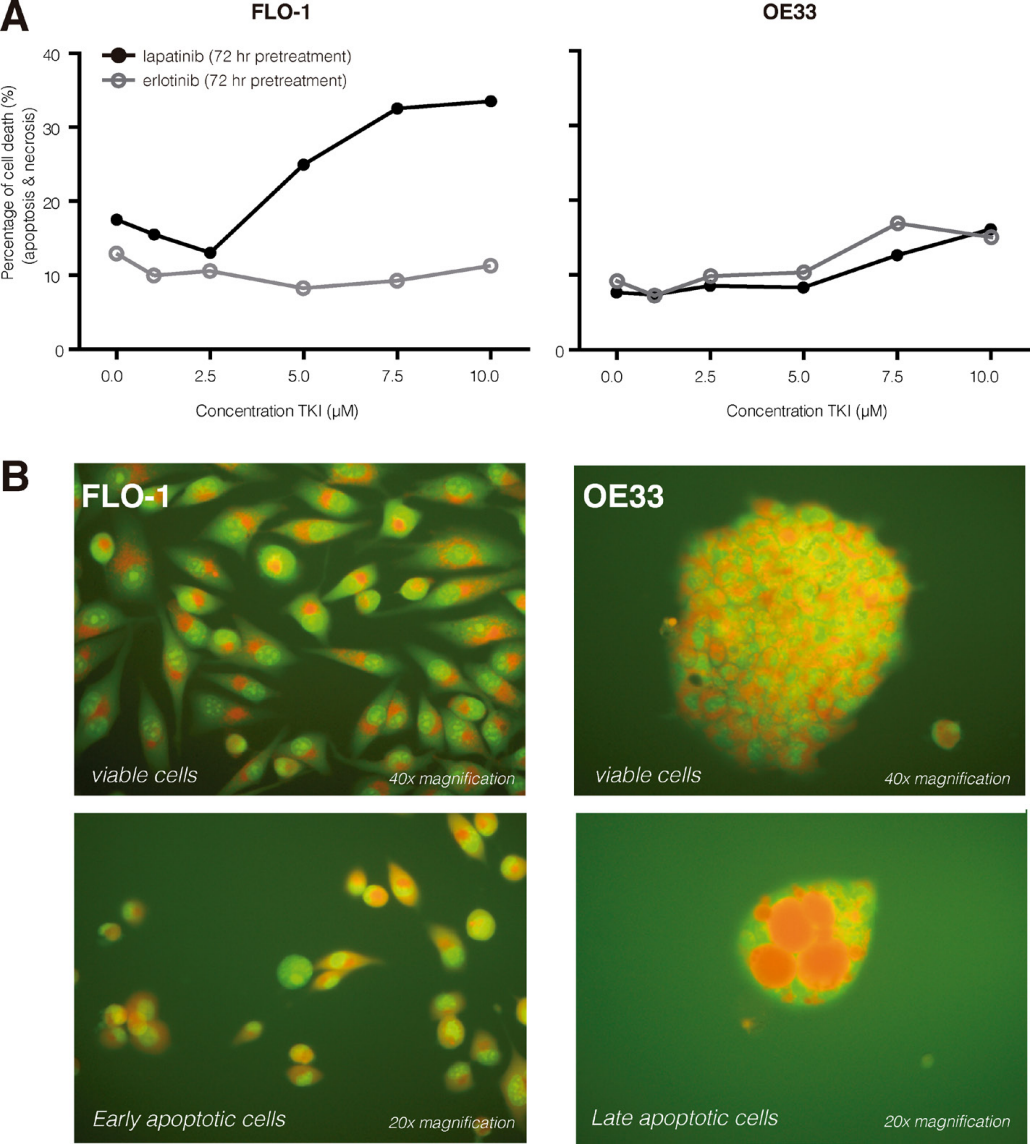
TKI induced cell death (**A**) Pretreatment with lapatinib (10 μM, 72 hr) induced apoptotic cell death (+10−30%) in both the FLO-1 and the OE33 cell lines. Pretreatment with erlotinib (10 μM, 72 hr) induced only negligible additional cell kill (+10%). (**B**) Using an AO/EB staining we demonstrate that TKIs induce apoptotic cell death; viable cells with intact membranes will have a uniform green color in their nuclei (upper row), early apoptotic cells show bright green patches in the nuclei (lower left) and late apoptotic cells have bright orange/red areas (lower right). Necrotic cells would have been uniform orange/red. AO/EB, acridine orange/ ethidium bromide. [* McGahon AJ, Martin SJ, Bissonnette RP, et al. The end of the (cell) line: Methods for the study of apoptosis *in vitro*. Methods Cell Biol. 1995;46:153–185].

We observed that pretreatment with 10 μM lapatinib induced apoptotic cell death (+10–30%) in both the OE33 and the FLO-1 cell lines (Figure 3). Pretreatment with 10 μM erlotinib induced only negligible additional cell kill (+10%). Finally, treatment with TKI for the duration of 72 hours also showed an observable growth inhibition as a treatment side effect in both EAC cell lines.

### Targeted photodynamic therapy (tPDT) using near-infrared (NIR) light

### Control experiments, blocking assay and baseline NIR-tPDT results

Our control experiments show that both components, NIR irradiation and the phototoxic compound, are necessary to accomplish treatment effects (Supplementary Figure 2A). In addition, adding 133nM unbound IRDye700DX, which is equivalent to the dye present in a conjugate dose of 10 μg/ml, showed only a minor nonsignificant increase in cytotoxicity upon irradiation compared to the untreated cells (+2%, *P* = 0.350; Supplementary Figure 2B). NIR-tPDT treatment with 10 μg/ml cetuximab-IRDye700DX resulted in significant more cytotoxicity compared to 133nM of unbound IRDye700DX (+2% vs. +54%, *P* = 0.017). Moreover, irradiation of cells treated with 10 μg/ml cetuximab-IRDye700DX (no wash; +54%) did not result in additional cell death in comparison to samples where the conjugate was washed away after incubation (+54%). Furthermore, to prove specificity of targeted cytotoxic conjugate, blocking assays were performed (Supplementary Figure 2C). We observed that after blocking of the EGFR binding sites with an excess of 1000 μg/ml cold cetuximab, only an insignificant amount of cell death occurred (+6%, *P* = 0.017), comparable to untreated cells.

The results of the baseline NIR-tPDT experiments in both EAC cell lines are presented in Supplementary Figure 3A; when applying cetuximab-IRDye700DX, we observed a stronger therapeutic effect in the OE33 cell line compared to FLO-1 cells (+20%). Trastuzumab- IRDye700DX was shown to be the most effective conjugate in the OE33 cells, since a plateau in cell death (±80%) was reached already at 20 J/cm^2^ compared to 40 J/cm^2^ for cetuximab-IRDye700DX. For both cell lines the percentages of cell death did not significantly differ between the three conjugate dosages tested (1 μg/μl, 5 μg/μl and 10 μg/μl of cetuximab-IRDye700DX or trastuzumab-IRDye700DX). The microscopic images presented in Supplementary Figure 3B demonstrate the effect of NIR-tPDT in the EAC cell lines; already after one hour following NIR-tPDT, both cell lines appeared to have a uniform orange/red colored nucleus, indicating rapid necrotic cell death.

### Receptor modulation significantly increases the effect of NIR-tPDT

**Figure 4:**
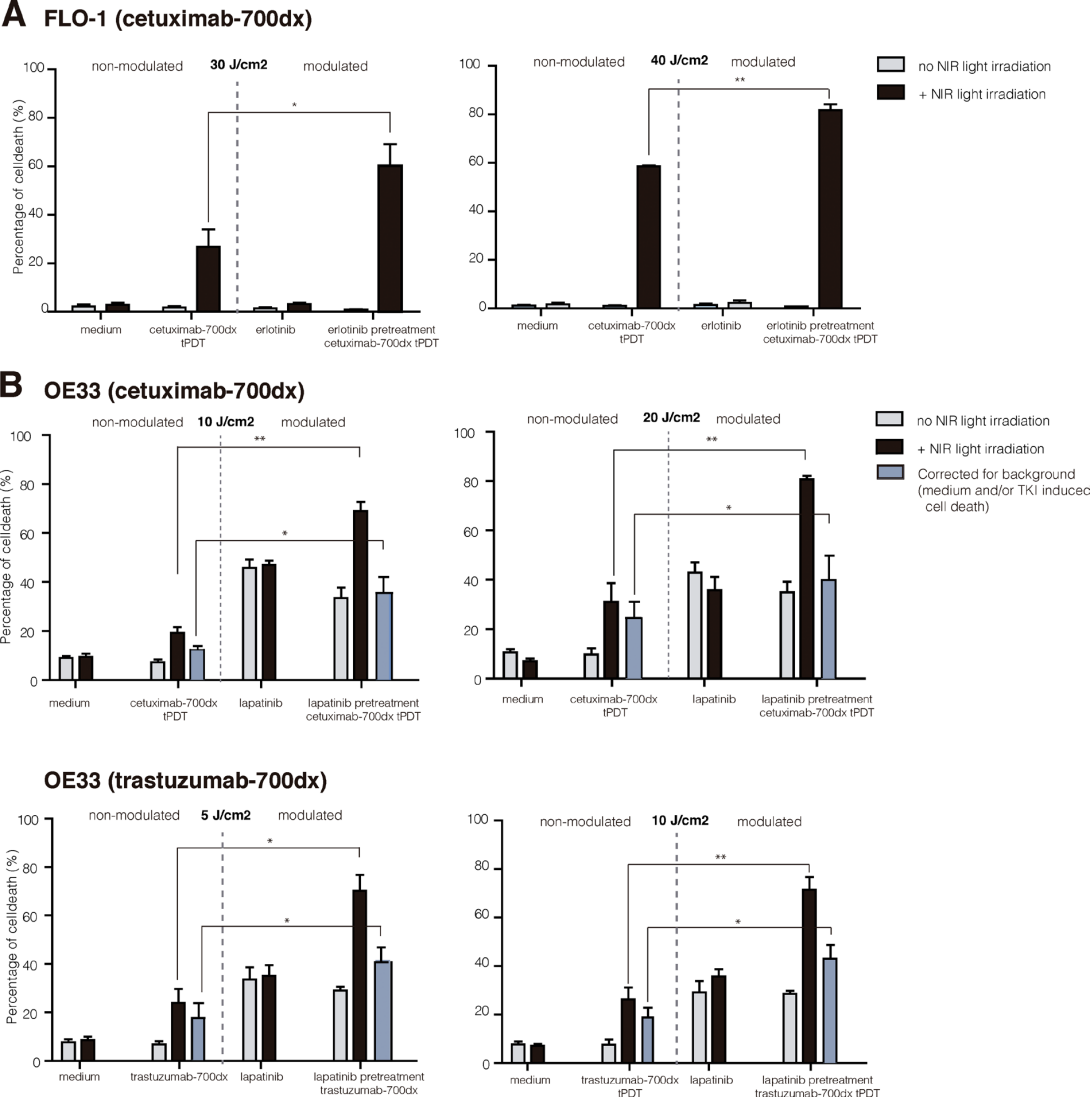
NIR-tPDT following EGFR/HER2 receptor modulation (**A**) In the FLO-1 cells, cetuximab-IRDye700DX NIR-tPDT following erlotinib pretreatment -EGFR upregulation- resulted in an additional 25% rapid, necrotic cell death. (**B**) In the OE33, lapatinib pretreatment (EGFR and HER2 upregulation) resulted in an increased therapeutic effect of both cetuximab-IRDye700DX (+50%) and trastuzumab-IRDye700DX (+45%) targeted NIR-tPDT. After correcting these results for the lapatinib induced cell kill (blue bar), the results remained significant (+24% additional cell kill). NIR-tPDT, near-infrared targeted photodynamic therapy; Results are presented using the mean (SD); *P < 0.005, **P < 0.0001.

In the FLO-1 cell line NIR-tPDT after erlotinib pretreatment resulted in significant additional cell death (+25%) (40 J/cm2 *P* = 0.001; 30 J/cm2 *P* = 0.044; Figure 4). We observed that the amount of cell death did not differ significantly between the varying NIR light dosages used (30–40 J/cm^2^). In the OE33 cells, NIR-tPDT after lapatinib pretreatment resulted in significant additional cell death when performing cetuximab-IRDye700DX (+50%) and trastuzumab–IRDye700DX (+45%) mediated NIR-tPDT treatment (Figure 4A trastuzumab 10 J/cm^2^ *P* = 0.001, 5 J/cm^2^ *P* = 0.002; cetuximab 20 J/cm^2^ *P* = 0.001, 10 J/cm^2^ *P* = 0.001). It should be noted that pretreatment of the OE33 cells with lapatinib already induced apoptotic cell kill (+10–30%). To still be able to evaluate the effect of NIR-tPDT, we corrected our results for this lapatinib-induced (‘background’) cell kill. After doing so, receptor modulation still resulted in significant additional cell death when treated subsequently with NIR-tPDT (trastuzumab: 5 J/cm^2^ +24% *P* = 0.032, 10 J/cm^2^+24% *P* = 0.013; cetuximab: 10 J/cm^2^ 36% *P* = 0.012, 20 J/cm^2^ 39% *P* = 0.026; Figure 4B).

## DISCUSSION

This *in vitro* study demonstrates the potential application of NIR-tPDT as treatment strategy in EAC. We furthermore show, as a first, that pretreatment of EAC cells with TKI -erlotinib or lapatinib- increases EGFR and/or HER2 receptor expression at the cell surface. By modulating the receptor status of EAC cells, we were able to enhance the treatment effects of EGFR/HER2-targeted NIR-tPDT. As such, we can conclude that NIR-tPDT seems a promising treatment option for esophageal adenocarcinoma and that TKI pretreatment is able to optimize the therapeutic effect of this novel cancer-selective treatment, making it interesting for future clinical translation.

In previous literature, some groups refer to NIR-tPDT as “near-infrared photo immunotherapy” (NIR-PIT); except for the terminology, the techniques are completely similar since both are IRDye700DX-based targeted PDT approaches. Over the last few years, the application of NIR-tPDT has been explored as a treatment option in several solid tumors; initially EGFR-targeting NIR-tPDT *in vitro* and *in vivo* experiments were performed using only the high EGFR-expressing epidermoid carcinoma cell line A431 [13, 25–31]. To date its use has been investigated in several other tumor types including glioblastoma, [32] ovarian cancer [33], HER2 negative and CD44-positive triple negative breast cancer [34–36], and head and neck cancer [37]. Besides, the first-in-human phase I tPDT trial in patients with inoperable head and neck cancer is currently recruiting patients (https://clinicaltrials.gov/ct2/show/NCT0242979). For the gastrointestinal tract, preclinical studies with gastric [38,39] and pancreatic cancer [40–42] have shown promising therapeutic responses to HER2 targeting NIR-tPDT treatment regimes. As such, we envision NIR-tPDT to be an effective approach in EAC as well. Especially the implementation of NIR-tPDT as previously described for pancreatic and head and neck cancer, where NIR-tPDT is applied to treat residual disease after surgical resection, could be a future application in EAC. For instance, NIR-tPDT following an irradical (incomplete) endoscopic mucosal resection might help to prevent highly invasive esophagectomy.

Our results demonstrate that NIR-tPDT is indeed an effective treatment strategy in EAC *in vitro*. The NIR-tPDT experiments performed in this study target two receptors of the EGF family, namely EGFR and HER2. The receptor expression results show that OE33 is a high expressing EGFR and HER2 cell line, which is in concordance with previous literature [43]. We observed that FLO-1 is a cell line with average expression of EGFR and low to negative expression of HER2; for the FLO-1 cells no results on receptor status have been reported thus far. During our baseline dose-escalation experiments, we concluded that the efficacy of NIR-tPDT was dependent mostly on the amount of NIR light exposure, and to a lesser extent on the conjugate dose used. This observation is in concordance with the results of Mitsunaga *et al*., who also concluded that the percentage of cell death in EGFR positive A431 cells was NIR light intensity dependent, and that repeated exposures of NIR light resulted in complete tumor responses [26].

NIR-tPDT is thought to be a cancer-selective therapy with minimal damage to surrounding healthy tissue [25,44]. Our study confirms this selectivity via a blocking assay, which resulted in a decrease of cell death after blocking of the EGFR binding sites with an excess of cold cetuximab. Furthermore, no significant cytotoxicity was observed in cells treated with only one of the components: NIR light or phototoxic conjugate. Previously, it was already demonstrated that EGFR targeted NIR-tPDT was only successful in EGFR positive cells by using a mixed tumor model that contained both receptor positive and negative cells [13,27]. Our results meet this assumption, since NIR-tPDT showed a higher cell kill in the OE33 cells compared to FLO-1; the baseline EGFR and HER2 receptor expression of the OE33 cells is considerably higher than that of the FLO-1 cells, implying its receptor selectivity. In addition, these results also imply that when more receptors are available at the cell surface, thus more binding sites for the phototoxic conjugate, this leads to more effective NIR-tPDT treatment. Our previous observation, that varying the NIR light intensity seems more effective than raising the conjugate dose to accomplish cell kill, might also be explained by the EGFR and HER2 receptor status; the amount of conjugate applied might already saturate all the available EGFR or HER2 receptors. As such, these results substantiate the hypothesis that receptor modulation could be a promising strategy for optimizing NIR-tPDT.

Previously, Shimoyama *et al*. explored viral transduction of the extracellular domain of an overexpressing growth factor receptor [35]. They employed virus mediated HER2 transduction in negative HER2 expressing breast cancer cells, with which they concluded that NIR-tPDT resulted in effectively and selectively killing of the HER2-transducted cells *in vitro*. However, viral transduction is not easily clinically translatable, whereas receptor modulation using clinically applicable TKI is. Based on these observations, we investigated the hypothesis that upregulation of the EGF receptors might enhance the treatment effect of NIR-tPDT. For the EAC cell lines used in our study, OE33 and FLO-1, no previous literature on TKI responsiveness was yet available; as such, we are the first to demonstrate that TKI administration is indeed able to significantly upregulate EGFR and HER2 receptor expression in EAC cells. More importantly, our results demonstrate that EAC cells, which already express the receptor to some extent, will gain additive effect from NIR-tPDT treatment after receptor modulation. Previous literature has demonstrated a positive effect of TKI pretreatment in conventional PDT [45–47], most likely caused by augmentation of the vascular effects of PDT and by improving photosensitizer uptake. Our study is the first to show the benefit of TKI-induced upregulation of target receptors and its positive effect on the NIR-tPDT treatment outcome.

In conclusion, this *in vitro* study demonstrates that EGFR or HER2 based NIR-tPDT is a promising treatment strategy in EAC. In addition, we demonstrated that pretreatment with the TKIs erlotinib and lapatinib induces significant growth receptor upregulation. This receptor upregulation resulted in an increased treatment response, making receptor modulation a promising strategy to optimize NIR-tPDT. Future *in vivo* experiments are required to evaluate whether clinical translation of NIR-tPDT combined with TKI pre-treatment is indeed feasible.

## MATERIALS AND METHODS

### Cell Culture

For our experiments we used four cell lines: OE33 (obtained from Dr. Bremer, dept. of Surgical Oncology, UMCG, Groningen, The Netherlands), FLO-1 (obtained from D. Beer, dept. of Surgery, university of Michigan, MI, USA), SW1573 and MCF-7 (both obtained from ATCC, LGC Standards, Middlesex, UK); all cell lines were tested and authenticated by BaseClear (BaseClear B.V., Leiden, The Netherlands).

The OE33 and FLO-1 are both human esophageal adenocarcinoma (EAC) cell lines. As positive growth receptor control cell lines we used SW1573, which is a low-expressing human non-small cell lung carcinoma cell line and MCF-7, which is an average EGFR-expressing human breast carcinoma cell line. The cells of the FLO-1 cell line were cultured in Dulbecco’s Modified Eagle Medium (DMEM); the other cell lines were cultured in Roswell Park Memorial Institute Medium (RPMI). All media were supplemented with 10% fetal calf serum (FCS), the medium of the SW1573 cell line also required glutamine (0.1%). Cells were cultured at 37°C, in a humidified atmosphere containing 5% CO_2_. Cells were passaged twice a week using trypsin (0.1%); both EAC cell lines were passaged at 1:6, MCF-7 at 1:10 and SW1573 1:20.

### Characterization of FLO-1 / OE33: baseline receptor expression analysis

To evaluate and quantify EGFR and HER2 expression levels at baseline, receptor expression analyses were performed using fluorescence-activated cell sorting (FACS; BD Accuri C6). Per experiment, approximately 400,000 cells were incubated with 0.625 μg of cetuximab (20 μg/ml; anti-EGFR) or trastuzumab (20 μg/ml; anti-HER2), for 1 hour at 4°C while on a shaker. Afterwards the cells were washed twice with PBS (1 ml, 2% FCS) and incubated with 1.1 μg of a secondary IgG anti-human monoclonal antibody labeled with fluorescein isothiocyanate (44 μg/ml; IgG-FITC, Sigma Aldrich). Incubation and washing of the secondary antibody was performed as described for the primary antibody. The relative mean fluorescence intensity (MFI) corrected for the background fluorescence signal (secondary antibody only), which was determined by using only the secondary antibody, was used as a measure of cell-surface receptor expression.

To quantify the receptor expression, we made use of the relative mean fluorescence intensity (MFI) measured with FACS analyses; to accomplish a reliable comparison between the different experiments and cell lines, the MFI of the OE33 cells was set at 100%, serving as a relative reference.

### EGFR and HER2 receptor modulation

### Receptor expression analysis following TKI treatment

To achieve receptor modulation, the cells were incubated in culture flasks with several concentrations [range: 1–10 μM] of erlotinib (anti-EGFR; stock solution 10 mM; LC Laboratories) or lapatinib (anti-EGFR and anti-HER2; stock solution 10 mM; LC Laboratories). After 24, 48 and 72 hours of TKI treatment, cells were harvested for FACS analysis. Since non-vital cells will detach, the medium of the treated cells was removed from the culture flasks but kept in a tube to prevent cell loss; attached cells were harvested using 0.5 ml trypsin per flask for 2–5 min. The cells were transferred into the tube with the already removed medium, spun (5 min, 1000 RPM, 4°C) and washed. Finally, the cells were resuspended in PBS (+2% FCS) and divided into tubes, each containing 200 μl.

The FACS analysis performed after 72 hours of treatment was combined with a cell death analysis. For this analysis, the cells were incubated with propidium iodide (PI), a marker for late apoptosis and necrosis; 1 μl PI was added per sample, incubated for 10–15 min on ice and FACS analysis was performed as described for the baseline expression. To quantify the amount of cell death during FACS analysis, gates were manually drawn around vital (P1) and dead (P2) cells. To calculate the percentage of cell death per sample, the amount of cells included in gate P2 was set against the total amount of cells (% cell death = (P2)/(P1 + P2)).

### Targeted photodynamic therapy (tPDT) using near-infrared (NIR) light

### Targeted phototoxic conjugates: cetuximab-IRDye700DX and trastuzumab-IRDye700DX

For the synthesis of the NIR-tPDT conjugates, cetuximab (Erbitux®, Merck B.V. Netherlands) and trastuzumab (Herceptin®, Roche) were used. Antibody samples were taken from the daily surplus of drugs used for clinical application by the hospital pharmacy (Department of clinical Pharmacy and Pharmacology of the UMCG). The buffers of cetuximab and trastuzumab were exchanged using a PD10 desalting column (GE Healthcare, Dublin, IRL), in order to remove histidine from the solution. For this step, we used a phosphate-based labeling buffer at pH 8.5 to provide optimal conjugation conditions after buffer exchange. The monoclonal antibodies were incubated with IRDye700DX NHS ester (LI-COR Biosciences, Lincoln, Ne, USA): 454.4 μg IRDye700DX (64.9 μl, 7 mg/ml, molar ratio 4:1) was added to 8.5 mg cetuximab (2.5 ml, 3.39 mg/ml) and 537.6 μg IRDye700DX (76.8 μl, 7 mg/ml molar ratio 4:1) was added to 10 mg trastuzumab (1.75 ml, 5.6 mg/ml). Dye-antibody mixtures were incubated at room temperature, shielded from light, for 120 minutes. The conjugates were analyzed for their concentration, purity, yield and labeling efficiency by use of size exclusion high performance liquid chromatography (SE-HPLC), which showed a label efficacy of 63% for cetuximab-IRDye700DX (average dye to protein ration 2.52:1) and 88% for trastuzumab-IRDye700DX (average dye to protein ration 3.51:1). The conjugates were purified with the PD-10 column, diluted to a stock-concentration of 1 mg/ml using 0.9% sodium chloride, and stored in the dark at 4°C.

### NIR-tPDT device

A previously described, an especially designed light emitting diode (LED) device (Phillips Consumer Lifestyle B.V., Eindhoven, NLD) was used to perform the NIR-tPDT experiments. [37,48] The device emits light at a peak wavelength of 690 nm, which is suitable for IRDye700DX excitation. In total, 126 individual LEDs emit NIR light, with an adjustable light intensity ranging from 41–206 mW/cm^2^. The energy of the NIR light source was calculated using the formula: *energy of the NIR light (mJ/cm*^2^*) = Intensity (mW/cm*^2^*) x Time (seconds)*. The required amount of NIR light energy was obtained by adjusting the light intensity (1–2.5 ampere; max. fluence rate 103 mW/cm^2^) and exposure time. Well plates were placed at a fixed distance of 20 cm from the light source.

### Control treatments and blockings assay

Control experiments were performed using cold cetuximab (10 μg/ml and 1000 μg/ml) and an equivalent of unbound IRDye700DX (133 nM) followed by NIR light irradiation with 0 J/cm^2^ and 2 J/cm^2^. Cells incubated with 10 μg/ml cetuximab-IRDye700DX and 1000 μg/ml cold cetuximab for 1 hour were used for blocking assay analysis, followed by NIR light irradiation with 0 J/cm^2^ and 2 J/cm^2^.

### NIR-tPDT experiments: baseline dose-escalation and after receptor modulation

The dose escalation was performed to determine the optimal NIR light and conjugate dose. Cells were seeded in 12-well plates (1 ml of standard medium per well) and grown for 24 hours. The medium of the wells was aspirated and the conjugate diluted in RPMI or DMEM was added. The cells were incubated for 1 hour at 37°C and the conjugate was removed. The wells were washed twice with PBS containing 2% FCS. Subsequently, the plates were irradiated using the LED device, performed in a dark room. The total light dose varied from 0 J/cm^2^ to 60 J/cm^2^; max. fluence rate: 103 mW/cm^2^). In addition, different dosages of conjugate (10–5-1 μg/ml) were tested at a fixed NIR energy (40 or 60 J/cm2; Supplementary Table 1). Per experiment, one plate was shielded from NIR light, serving as the negative NIR irradiation control. Moreover, all plates were shielded from ambient light throughout the experiment. After NIR-tPDT, FACS analysis was performed to evaluate cell death.

Similar NIR-tPDT experiments were performed following EGF-receptor modulation. After seeding the cells and 24 hours of cell growth, one of the two TKIs was added to the 1 ml medium volume of the 12-well plates and incubated at 37°C for 72 hours. Thereafter the NIR-tPDT protocol, as described above, was executed for the pretreated cells using various NIR light amounts (Table 1).

**Table 1:** Dosage of NIR-tPDT following receptor up-regulation

Cell line	Conjugate	Energy of NIR light
OE-33	10 μg/ml cetuximab-IRDye700DX	10 J/cm^2^ - 20 J/cm^2^
	10 μg/ml trastuzumab-IRDye700DX	5 J/cm^2^ - 10 J/cm^2^
FLO-1	10 μg/ml cetuximab-IRDye700DX	30 J/cm^2^ - 40 J/cm^2^

### Comprehensive cell death analysis: acridine orange/ ethidium bromide staining (AO/EB)

To more extensively analyze the type of cell death that occurs following TKI treatment and NIR-tPDT, an additional and more comprehensive cell death analysis was performed: the *acridine orange/ ethidium bromide* (AO/EB) cell death analysis. Acridine orange (AO) intercalates into DNA and is a marker for early apoptosis. Ethidium bromide (EB) is only taken up by non-viable cells and is a marker for late apoptosis and necrosis. To analyze the type of cell death due to TKI treatment, the cells were cultured in a 96-well plate with increasing concentrations of lapatinib and erlotinib in 200 μl of culture medium. After 24 and 48 hours of incubation, 1 μl of AO (0.5 mg/ml; Sigma) and 1 μl of EB (1 mg/ml; Invitrogen, Paisley) were added to the wells. After 10 min of incubation, the cells were spun (5 min, 1000 RPM, 21°C) and supernatants were discarded. For the NIR-tPDT treated cells the same amounts of AO/EB was added, but then within one hour after the tPDT treatment was finalized. Finally, the cells were viewed under the microscope and the percentage of apoptotic or necrotic cells was calculated.

### Statistical analyses

All presented results are derived from at least three independent experiments. The data is analyzed with SPSS version 23.0 and Graphpad Prism version 5.04 and presented as the mean and standard deviation (SD). The differences between the various treatment groups within the receptor modulation experiments was analyzed using a Kruskal Wallis test with a post-hoc Holms-Bonferroni correction for multiple testing. The effects of additional cell death after receptor modulated NIR-tPDT was analyzed using an independent, two-sided, *T-test*. The global significance level was set at *P* < 0.05.

## SUPPLEMENTARY MATERIALS FIGURES AND TABLES



## Abbreviations

EC: Esophageal cancer
SCC: Squamous cell carcinoma
EAC: esophageal adenocarcinoma
GERD: gastroesophageal reflux disease
PDT: photodynamic therapy
tPDT: targeted photodynamic therapy
NIR: near-infrared
mAB: monoclonal antibody
EGF: epidermal growth factor
FACS: fluorescence-activated cell sorting
