# Supplementary material for: Tyrosine kinase inhibitor induced growth factor receptor upregulation enhances the efficacy of near-infrared targeted photodynamic therapy in esophageal adenocarcinoma cell lines
Source: Oncotarget. 2017 Mar 13;8(18):29846–56. doi: 10.18632/oncotarget.16165 (PMC5444708; doi:10.18632/oncotarget.16165)
Supplement: Supplementary file 1 [file oncotarget-08-29846-s001.pdf]

Tyrosine kinase inhibitor induced growth factor receptor upregulation enhances the efficacy of near-infrared targeted photodynamic therapy in esophageal adenocarcinoma cell lines

Supplementary Materials

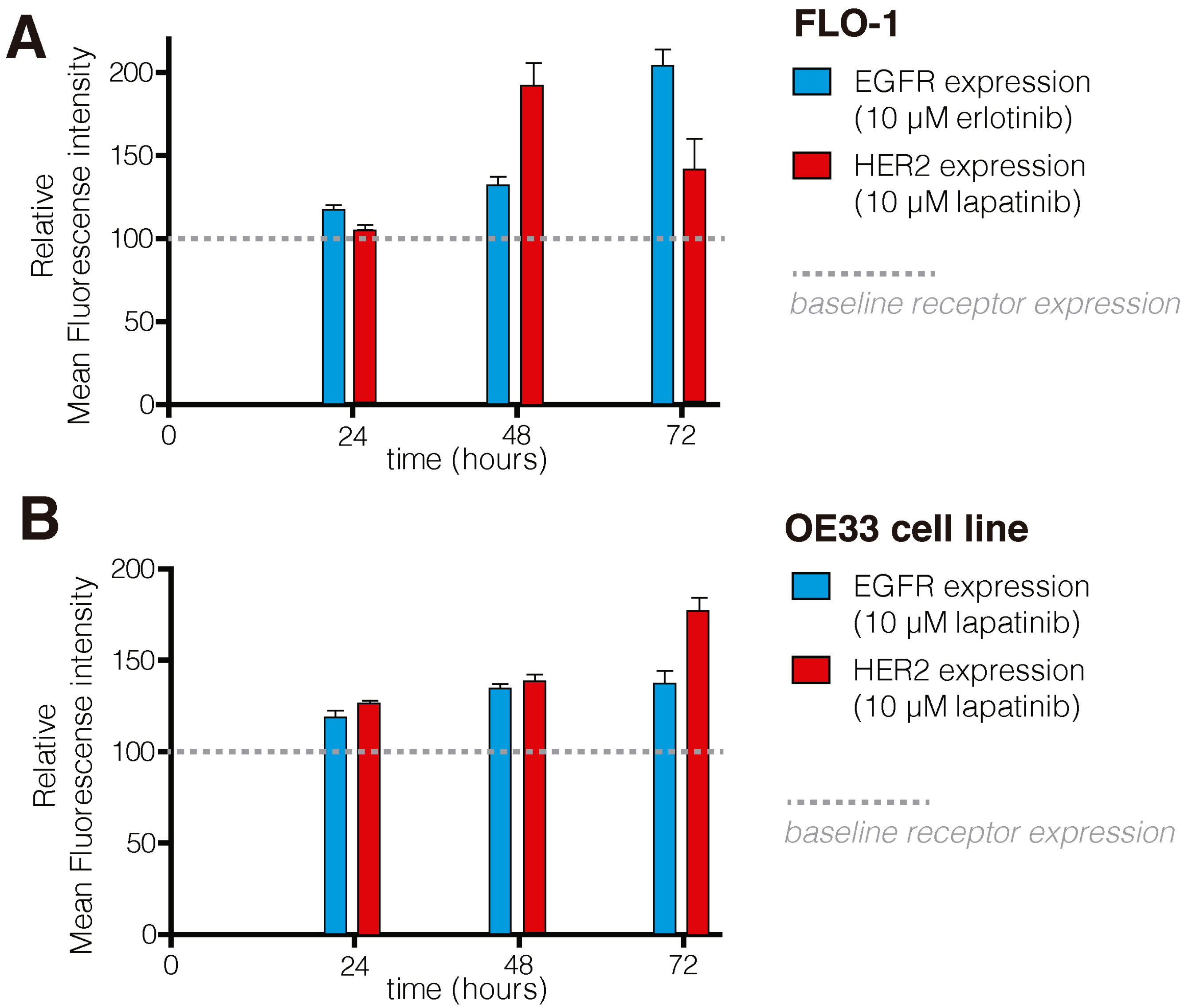

Supplementary Figure 1: EGFR and HER2 upregulation over time. Overall, 72 hours of TKI pretreatment was found to be the most effective for receptor upregulation. (**A**) Following 72hrs. of erlotinib pretreatment, the EGFR receptor in the FLO-1 cells doubled (+104%). The HER2 receptor also showed an increase, though with a maximum at 48 hrs. (+93%), which dropped when treatment continued. (**B**) For the OE33 cells, 72 hrs. of lapatinib pretreatment resulted in an increase of both the EGFR (+37%) and HER2 receptor (+78%). Since erlotinib pretreatment in the OE33 resulted in downregulation of EGFR, this TKI was not more extensively tested over time.

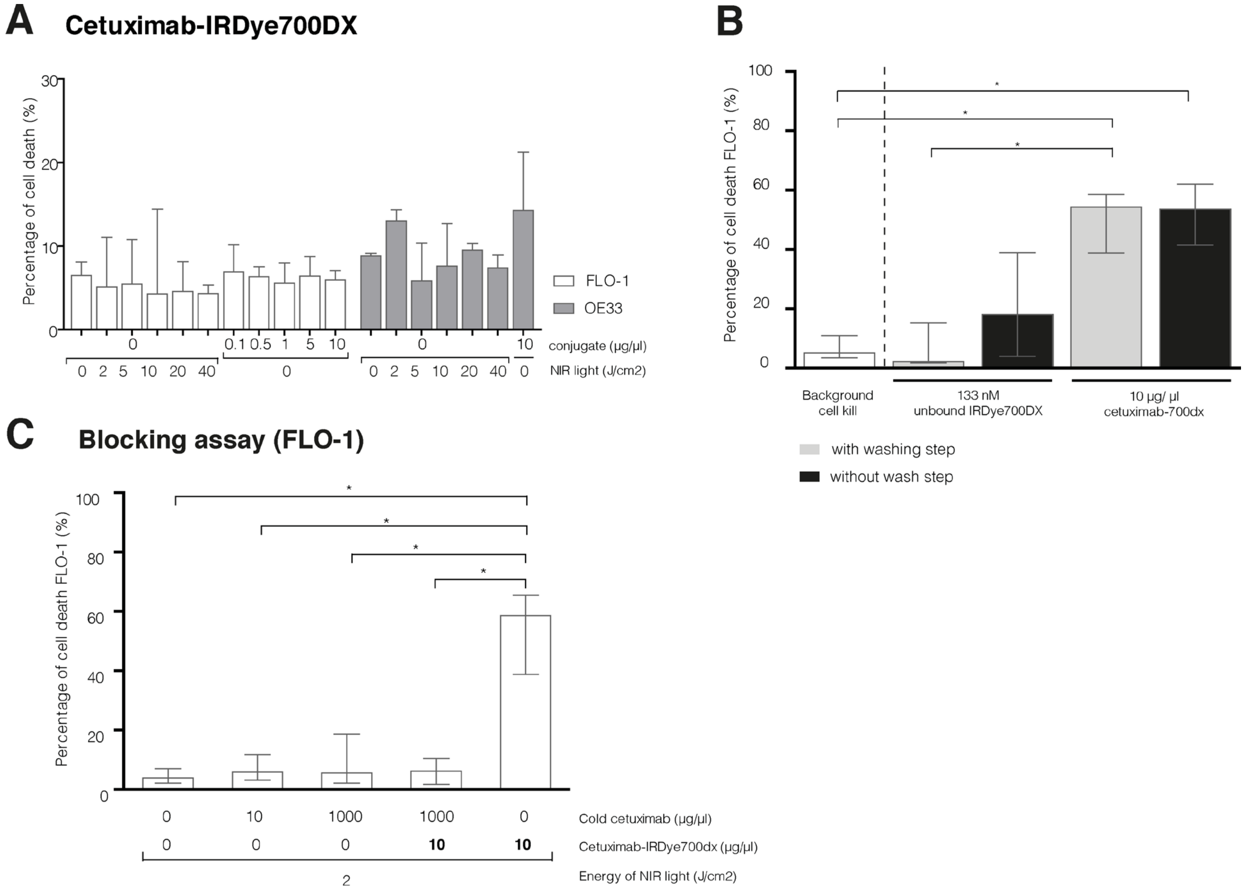

Supplementary Figure 2: Cetuximab-IRDye700DX control experiments and blockings assay. (**A**) Only minimal cell death is observed in the cells which received only one of the NIR-tPDT components: either cetuximab-IRDye700DX or NIR light irradiation. (**B**) NIR irradiation of samples with unbound IRDye 700DX or an excess of cetuximab-IRDye700DX (without washing step) does not lead to additional cell death. (**C**) Target-specific cell death is confirmed by a significant decrease in percentage of cell death after blocking EGFR with cold cetuximab in FLO-1 cells. Cell death in FLO-1 cells treated with cetuximab-IRDye700DX followed by NIR light irradiation is significant higher in comparison to cells that are only irradiated or cells treated with cold cetuximab followed by irradiation. REsults are presented using the mean (SD). *P 0.05. NIR-tPDT, near-infrared targeted photodynamic therapy.

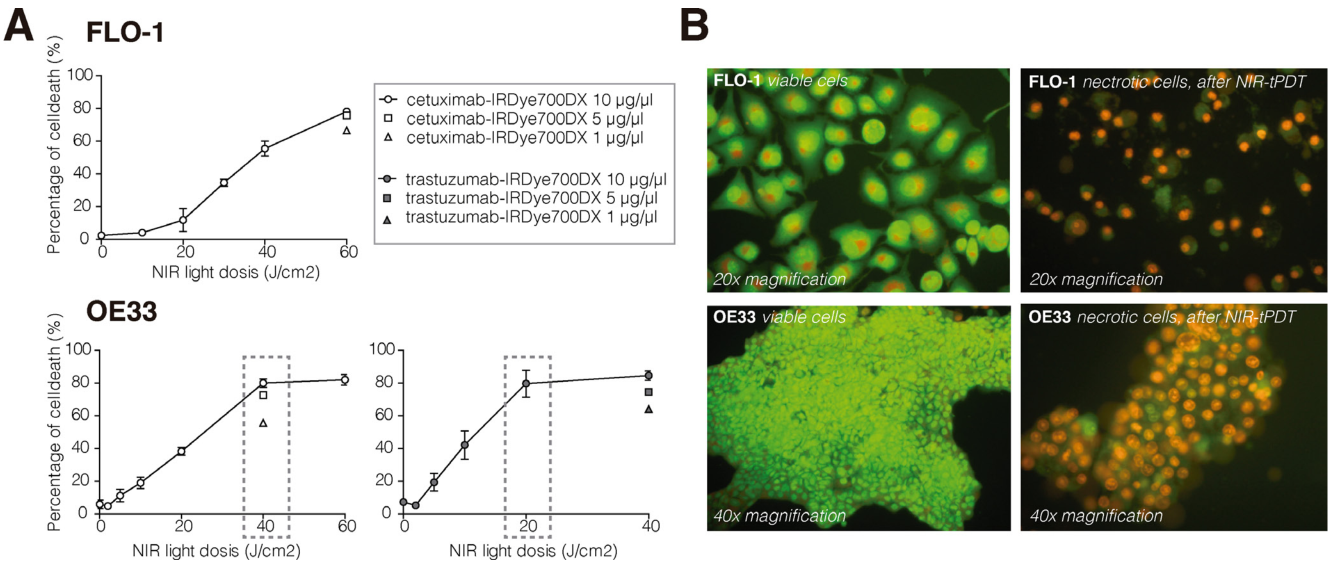

Supplementary Figure 3: Baseline NIR tPDT results. (**A**) The results of the baseline NIR-tPDT experiments in both EAC cell lines are presented; when applying cetuximab-IRDye700DX, we observed a general stronger therapeutic effect in the OE33 cell line compared to FLO-1 cells (+ 20%). Trastuzumab- IRDye700DX showed to be the most effective conjugate in the OE33 cells, since a plateau in cell death (± 80%) was reached already at 20 J/cm2 (dashed grey rectangle). For both cell lines the percentages of cell death did not significantly differ between the three conjugate dosages tested [1, 5,10 µg/µl]. (**B**) The microscopic images show that already one hour after NIR-tPDT treatment cells appeared to have an uniform orange/red colored nucleus, indicating rapid necrotic cell death. *[McGahon AJ, Martin SJ, Bissonnette RP, et al. The end of the (cell) line: Methods for the study of apoptosis in vitro. Methods Cell Biol. 1995;46:153-185].*

Supplementary Table 1: Dosages of conjugates and energy of NIR light for the dose escalation experiments

Cell line	Conjugate	Energy of NIR light	Dosages of conjugate
OE-33	cetuximab-IRDye700DX	0-5-10-20-40-50 J/cm^2	40 J/cm^2: 10−5−1 µg/ml conjugate
	trastuzumab-IRDye700DX	0-2-5-10-20-40 J/cm^2	40 J/cm^2: 10−5−1 µg/ml conjugate
FLO-1	cetuximab-IRDye700DX	0-10-20-30-40-50 J/cm^2	60 J/cm^2: 10−5−1 µg/ml conjugate
